# Identification of immunostimulatory activities and active compounds from sequentially extracted fractions of rhizosphere fungal fermentation broth of *Atractylodes macrocephala* Koidz. rhizomes

**DOI:** 10.3389/fphar.2024.1460614

**Published:** 2024-12-20

**Authors:** Yuxin Xie, Na Lin, Pingping Song, Xiangyan Ni, Yakun Wang, Peng Huang, Zhili Han, Dianlei Wang, Nianxia Sun

**Affiliations:** ^1^ College of Pharmacy, Anhui University of Chinese Medicine, Hefei, China; ^2^ Beijing Municipal Bureau of Agriculture and Rural Affairs, Beijing Agricultural Product Quality and Safety Center, Beijing, China; ^3^ Anhui Province Key Laboratory of Research and Development of Chinese Medicine, Hefei, China

**Keywords:** *Atractylodes macrocephala* Koidz. rhizome, rhizosphere fungi, immunological activity, secondary metabolites, RAW 264.7 cells, UHPLC-Q-TOF-MS

## Abstract

**Introduction:**

Pharmacological studies have shown that the rhizome of Atractylodes macrocephala Koidz. (Compositae), commonly known as atractylodes macrocephala rhizome (AMR), can modulate immunity. Nevertheless, its resources have been largely depleted, and the pharmacological activity of artificial AMR is relatively modest. We hypothesized that the fermented crude extracts of the rhizosphere fungi of AMR would have similar immunomodulatory effects since the metabolites generated by these fungi are similar to those of the host plant given their long-term synergistic evolution.

**Methods:**

Rhizosphere fungi were isolated from the rhizosphere soil of AMR and cultured to produce the secondary metabolites. These metabolites were then sequentially extracted with four solvents of increasing polarities (petroleum ether, ethyl acetate, n-butanol, and water). The in vitro immunomodulatory activities of the metabolite extracts were evaluated by cell proliferation capacity, cell phagocytosis activity, NO secretion capacity, cell morphology changes, and cytokine (TNF-α, IL-1β and IL-6) secretion capacity in RAW264.7 macrophage cells. The biologically active secondary metabolites produced by the rhizosphere fungi were identified using ultrahigh-performance liquid chromatography coupled with quadrupole time-of-flight mass spectrometry (UHPLC-Q-TOF-MS).

**Results:**

Three rhizosphere fungi, namely Penicillium (MK-1), Penicillium glaucoroseum (MN-1), and Purpureocillium lilalium (MG-1), were isolated from the rhizosphere soil of AMR. The assays for cell proliferation capacity, cell phagocytosis activity, and NO secretion capacity showed that all metabolite extracts exhibited in vitro immunomodulatory activities. The crude extracts of MG-1 exhibited the highest levels of in vitro immunomodulatory activities compared to the other extracts. Furthermore, it was demonstrated that the fermented extracts of MG-1 could facilitate immunological enhancement in vitro by altering the cellular morphology in the resting state and increasing the secretions of TNF-α, IL-1β, and IL-6. Meanwhile, there was no observable endotoxin contamination. The metabolite profiling of MG-1 by UHPLC-Q-TOFMS revealed the presence of several compounds with established immunoreactive activities, including L-arginine, prostaglandin I2, deoxyguanosine, bestatin, and osthole.

**Discussion:**

The present study demonstrated that the metabolite extracts of the rhizosphere fungi isolated from the rhizosphere soil of AMR exhibited in vitro immunoreactive activities and that these rhizosphere fungi could produce several bioactive metabolites. The crude extracts of the rhizosphere fungi may hence extend the medicinal utility of AMR and provide a basis for further development of natural plant-based immunomodulators.

## 1 Introduction

Immunity refers to the ability of the body to fend off invasion by pathogenic microorganisms and various diseases. Macrophages are the first line of defense for recognizing invading pathogens. When the body is threatened by foreign substances, infectious microorganisms, or cancer cells, macrophages can mount an effective defense through the processes of phagocytosis as well as secretion of cytokines and cytotoxic molecules, such as tumor necrosis factor (TNF)-α, interleukin (IL)-1β, IL-6, and nitric oxide (NO) (Wang et al., 2024). Accumulating evidence suggests that numerous plants and microbes, such as golden root (*Rhodiola rosea* L.) and filamentous fungi, possess potent immunomodulatory activities (Pu et al., 2020; Sutter et al., 2016).

Atractylodes macrocephala rhizome (AMR) refers to the desiccated rhizomes of *Atractylodes macrocephala* Koidz. (Compositae), which is a traditional Chinese herb used in tonics and a functional food ingredient. It is a superior-quality product noted in “Sheng Nong’s Classic of Materia Medica” (A.D. 25–220) that contains a considerable number of active ingredients, including polysaccharides, lactones, volatile oils, and amino acids. It has been described in “Ben Cao Qiu Zhen” (A.D. 1644–1911) as “the prime medicine for invigorating the spleen and for supplementing qi (vital energy).” As the most important secondary lymphoid organ, the spleen exerts an undeniable influence on the immune process, often carrying out its tasks through phagocytosis and immune reactions (Baldissera et al., 2017; Sang et al., 2013; Singh et al., 2024; Zhu et al., 2018). In “Yao Xing Lun” (A.D. 618–907), AMR has been indicated for the treatment of gastric discomfort, alleviation of cold and fevers, and stabilization of the fetus. In traditional Chinese medicine theory, these diseases are postulated to be associated with immunity. Modern pharmacological studies have shown that AMR can regulate immunity (Xu et al., 2012; Jun et al., 2018; Kwak et al., 2018; Li et al., 2009; Sun et al., 2011). AMR from Qimen (Anhui, China) is a rare and authentic medicinal material with superior pharmacological activity and chemical composition compared to other sources (Cui et al., 2020). A local proverb from Qimen states “A serious illness, Qi do not leave, eat two catties, uphill strength.” However, AMR from Qimen has specific requirements in terms of the growth environment and was only found to be distributed on the foothills of mountains with fertile soil at an altitude of 1000 m. Therefore, its distribution in the natural biological setting is incredibly rare. The AMR resources from Qimen have largely declined owing to its slow growth rate and overexploitation. Despite attempts at artificial introduction, the chemical composition and pharmacological activity were found to be comparatively poor.

The emphasis in drug research has gradually shifted from pharmaceutical plants to microorganisms. To date, more than 20,000 metabolic products of microorganisms, particularly those that are physiologically active, have been characterized (Katoch et al., 2014). Penicillin and cyclosporine, which exhibit bactericidal and immunosuppressive activities, respectively, are isolated from *Penicillium sp.* and *Tolypocladium inflatum* Gams (Liddicoat and Lavelle, 2019; Monteillier et al., 2018). Compared to plant resources, microorganisms have the advantages of easy proliferation and possibility for industrial production under controlled conditions. The plant rhizosphere is a complex ecosystem comprising a multitude of beneficial microorganisms (Badri et al., 2009; Butler et al., 2003). Rhizosphere fungi are important components of plant rhizosphere microecosystems and exhibit a remarkable diversity of secondary metabolites as well as large enzyme-producing systems. These fungi exhibit potential pharmacological activities, such as hypoglycemic (Martel et al., 2016), antiproliferative, antiaging, anti-inflammatory (Pan et al., 2021), anticarcinogenic (Maheshwari et al., 2017; Niazi et al., 2023; Sahu et al., 2020), cholesterol-lowering (Nicoletti et al., 2007), antifungal (Nishihara et al., 2000; Tistechok et al., 2023), antiviral (Nishihara et al., 2000), and antioxidant (Martel et al., 2016; Sahu et al., 2020) properties.

In a previous study, we found that there were significant differences in the composition, diversity, and functionality of the rhizosphere microbial community between the AMR from Qimen and captive AMR. The AMR from Qimen and its rhizosphere fungi had formed a highly exclusive symbiotic mechanism, showing their specificity in different varieties of the same plant (Song et al., 2023). The rhizosphere fungi had an alternate relationship with the host plant, where the host plant provided a stable growth environment and rich nutrition for the growth of the fungi. Simultaneously, some rhizosphere fungi were observed to facilitate plant growth and enhance the accumulation of secondary metabolites (Zhou et al., 2018; Zhai et al., 2016). As a consequence of long-term synergistic evolution, the metabolites produced by the rhizosphere fungi were found to be similar to those of the host plant; thus, we hypothesized that the fermented crude extracts of the rhizosphere fungi of AMR from Qimen could also have immunomodulatory effects. Accordingly, the present study aimed to screen the rhizosphere fungal strains with high immune efficacies *in vitro* and trace their bioactive components derived from the AMR from Qimen with the objective of providing new resources for the development of immune-active substances.

## 2 Materials and methods

### 2.1 Chemicals and reagents

Ethyl acetate, n-butanol, and sodium hypochlorite were provided by Macklin Biochemical Co. (Shanghai, China). Fetal bovine serum (FBS) was provided by Wisent Biotechnology Co. (Jiangsu, China). The cytokine assay kits, trypsin solution, and NO assay kit were provided by Meimian Industrial Co. (Jiangsu, China). Dulbecco’s modified eagle medium (DMEM) was provided by Procell Life Science and Technology Co. (Wuhan, China). Cell counting kit-8 (CCK-8), lipopolysaccharides (LPSs), and neutral red solution (0.1%) were provided by Biosharp. The Gel Clot TAL Endotoxin Test kit was provided by Bioendo Technology Co. (Xiamen, China). The purities of the solvents and reagents used in the research were all at the analytical levels.

### 2.2 Extraction method

#### 2.2.1 Rhizosphere soil collection

The rhizosphere fungi were isolated from wild AMR rhizosphere soil samples in Qimen County, Huangshan City, Anhui Province (N29·35′, E117·57′). Dr. Wang (Department of Pharmacognosy, Anhui University of Chinese Medicine, China) was invited to identify the plant samples. Furthermore, the plant name was verified through consultation with “The Plant List” (www.theplantlist.org). Root samples from healthy AMRs were collected by removing the humus from the soil and then digging vertically along the roots to a depth of 0–20 cm. The non-rhizosphere soil attached to the roots was removed manually by gently tapping it off. The residual soil, situated approximately 2 mm from the roots, was collected and transferred to sterile plastic bags for utilization in the experiments (Song et al., 2023; Liu et al., 2024).

#### 2.2.2 Isolation and storage of the rhizosphere fungi

The method reported by Fuentes-Quiroz et al. (2023) was employed for the stepwise isolation of the rhizosphere fungi on potato dextrose agar (PDA) medium. The medium consisted of 200 g of potato (peeled), 20 g of dextrose, 20 g of nutrient agar, and 1,000 mL of distilled water. In brief, the method used was as follows: 1 g of the soil sample and 9 mL of sterile water were mixed, and serial dilutions were prepared to obtain different concentrations of the soil (Wang et al., 2023). Soil dilutions of varying concentrations were then inoculated onto the PDA medium and incubated in a constant-temperature incubator. Representative fungi were then selected and streaked on a newly configured PDA medium before incubation under the aforementioned conditions. These fungi were stored in freezing tubes containing 1,500 µL of glycerol (30%) and preserved at −20°C.

#### 2.2.3 Identification of the rhizosphere fungi

Prior to molecular identification of the rhizosphere fungi, the isolated fungal samples were screened on the basis of macroscopic phenotypic characteristics (Mazumder et al., 2021). The fungal total DNA was obtained using a fungal genomic DNA extraction kit. The polymerase chain reaction (PCR) amplification method with internal transcribed spacer (ITS) universal primers was employed to amplify the ITS region of the rhizosphere fungi (Chauhan et al., 2022). ITS1-forward primer (5′-TCC​GTA​GGT​GAA​CCT​GCG​G-3′) and ITS4-reverse primer (5′-TCC​TCC​GCT​TAT​TGA​TAT​GC-3′) were employed. The following conditions were employed for the PCR amplification: original denaturation at 94°C for 10 min; denaturation at 94°C for 30 s, annealing at 55°C for 20 s, and elongation at 72°C for 15 min for 40 cycles; final elongation at 72°C for 10 min. Then, approximately 50 μL of the reaction solution was prepared containing 1 μL of the amplified DNA, 25 μL of 2× Tsing KE Master Mix, 22 µL of dH_2_O, and 1 µL of each primer (10 µM). The fungal identities were determined primarily by ITS sequence alignment using the basic local alignment search tool in the National Center for Biotechnology Information Genbank database.

#### 2.2.4 Fermentation and extraction of the rhizosphere fungal metabolites

Vigorously growing mycelia were selected and inoculated aseptically in a potato glucose broth medium at 28°C for 14 days. Then, the liquid components were retained, while the solids were homogenized and extracted using acetone for 30 min. The extract was combined with the retained liquid, and the secondary metabolites were sequentially extracted thrice using four solvents of increasing polarities (petroleum ether, ethyl acetate, n-butanol, and water) at 1:1 volume at ambient temperature. The upper non-aqueous phase was subsequently separated and concentrated under reduced pressure to yield the petroleum ether, ethyl acetate, n-butanol, and water extracts. Because of their low compositions, the water and petroleum ether extracts are not discussed further in this article.

### 2.3 Evaluation of *in vitro* immunological activities

#### 2.3.1 Cell culture

RAW 264.7 cells, which are macrophages, were provided by the Shanghai Institutes for Biological Sciences (Shanghai, China). These cells were observed to remain active in the incubator under the DMEM system comprising 10% FBS, 100 U/mL of penicillin, and 100 μg/mL of streptomycin.

#### 2.3.2 Determination of cell viability

The CCK-8 kit was used to determine the efficacies of the fermented crude extracts of the rhizosphere fungi on the proliferative activity of the macrophages (Li et al., 2024). Accordingly, 96-well plates (5 × 10^4^ cells/well) were inoculated with 100 μL of the fungal-extract-treated RAW 264.7 cells at concentrations of 5, 10, 20, 40, 80, and 160 μg/mL. The positive and negative controls were implemented using LPS solution (2 μg/mL) and serum-free DMEM, respectively. After 24 h of incubation in the incubator, a 20 μL supplement of CCK-8 solvent was added and the mixture was incubated with shaking for 1 h. An enzyme marker (Thermo Fisher Scientific, United States) was then utilized to determine the optical density data at 450 nm from each well.

#### 2.3.3 Determination of phagocytic activity

The neutral red method was used to assess the phagocytic activities of the fermented crude extracts of the rhizosphere fungi on RAW 264.7 cells (Ates et al., 2017). Here, 96-well plates (5 × 10^4^ cells/well) were inoculated with 100 μL of the fungal-extract-treated RAW 264.7 cells. The positive and negative controls were established through the addition of LPS solution (2 μg/mL) and serum-free DMEM, respectively. After 24 h of incubation in the incubator, the medium was carefully discarded, the samples were washed thrice with phosphate-buffered saline (PBS) solution, and neutral red solvent (100 μL, 0.1%) was added to each well before further incubation. Following aspiration and disposal of the neutral red solution, a cell lysate reagent (1% acetate + 49% PBS + 50% ethanol) was added and shaken for 30 min. Lastly, an enzyme marker (Thermo Fisher Scientific, United States) was used to analyze the optical density at 540 nm from each well.

#### 2.3.4 Determination of NO

The NO assay kit was employed to assess the impacts of the fermented crude extracts on NO secretion in the RAW 264.7 cells (Marrocco and Ortiz, 2022). Here, the experimental group was treated with 100 μL of each fungal extract. After incubation, an enzyme marker (ThermoFisher, United States) was used to analyze the optical density of a solution comprising the cell supernatant and Griess reagent in a 1:1 ratio at 540 nm from each well.

### 2.4 Effects of *Purpureocillium lilalium* (MG-1) crude extracts on the morphologies and cytokine secretions of RAW 264.7 cells

#### 2.4.1 Determination of cellular morphology

Given the elevated immune activities observed, MG-1 was subjected to further investigation. The RAW 264.7 cells were inoculated onto 96-well plates (5 × 10^5^ cells/well). The cells in the experimental group were then treated with 200 μL of MG-1 fungal extracts at various concentrations (5, 10, 20, 40, and 80 μg/mL). The positive and negative groups were treated with LPS solution (2 μg/mL) and serum-free DMEM, respectively. A fluorescent inverted microscope (Olympus, Japan) was used to analyze the morphological variations after 24 h of incubation.

#### 2.4.2 Determination of cytokine secretions

The experimental groups, including the negative and positive controls, were prepared according to the above treatments. The cell supernatant was then collected and cytokine content was measured using the ELISA reagent kit. An enzyme marker (ThermoFisher, United States) was then used to analyze the absorbance at 450 nm for each well.

#### 2.4.3 Determination of endotoxin contamination

Endotoxin contamination of the fermented extracts was tested using the Gel Clot TAL Endotoxin Test kit (Xiamen, China). Two distinct concentrations of the LPS solution (150 and 300 μg/mL) and water (0.1 mL) were used here for the positive and negative groups, respectively. The experimental group was supplemented with different extraction solutions (100 μL, 2 mg/mL). In brief, all samples were incubated with the limulus amebocyte lysate reagent; if a gel had formed and the mixture remained intact at the bottom of the tube, the test was deemed positive.

### 2.5 Identification of bioactive metabolites in the fermented crude extracts of MG-1

The bioactive metabolites in the MG-1 extracts were characterized via ultrahigh-performance liquid chromatography coupled with quadrupole time-of-flight mass spectrometry (UHPLC-Q-TOF-MS; Triple TOF 6600, AB Sciex, UHPLC-Q-TOF-MS system). The samples were separated using an Acquity UPLC BEH amide column (1.7 µm, 100 × 2.1 mm, Waters, United States, 0.5 mL/min, 25°C). The mobile solvent system consisted of a solution of ammonium acetate and ammonium hydroxide in water (mobile phase A) and acetonitrile (mobile phase B). The samples were eluted using the following linear gradients: 0–0.5 min, 100%–5% of A; 0.5–7 min, 5%–35% of A; 7–8 min, 35%–60% of A; 8–9 min, 60% of A; 9–9.1 min 60%–5% of A; 9.1–12 min, 5% of A. The total volume of sample collected was 2 μL. The mass range was configured as 60–1,000 mass-to-charge ratio (M/Z), with the ion source gas1 and gas2 both set to 60 psi and a temperature of 600°C. The ion source spray voltage was set to ± 5,500 V, collision energy was 35 V ± 15 eV, declustering potential was ± 60 V, and TOF-MS scan frequency was set to 0.20 s/spectrum. The data were assessed and validated using the Agilent Masshunter Software and Metlin Metabolite database, with particular focus on the M/Z value, fragment ions, and retention time.

### 2.6 Statistical analysis

The data were subjected to statistical analysis with SPSS 23.0 software, and the results were expressed in their final forms as mean ± standard deviation (SD) (n ≥ 3). To ascertain the differences, we used one-factor ANOVA, Tukey’s range test, and Dunnett-t test. The findings were considered to be significantly different for *p* < 0.05.

## 3 Results

### 3.1 Fungal isolation and identification

Through morphological analysis and ITS sequencing, three rhizobial strains were separated from the rhizosphere soil of AMR and identified in this study. As illustrated in Table 1, these three rhizosphere fungi were Penicillium (MK450684.1), *Penicillium glaucoroseum* (MN864187.1), and *Purpureocillium* (MG148343.1), which we designated as MK-1, MN-1, and MG-1, respectively, by comparing the ITS sequences. MK-1 and MN-1 isolated in this study were identified as belonging to the *Aspergillaceae* family and *Penicillium* genus, while MG-1 was determined to belong to the *Ophiocordycipitaceae* family and *Purpureocillium* genus.

**TABLE 1:** Sequence similarities of rhizosphere fungi isolated from AMR with the sequences registered in GenBank.

Isolated fungal sequence		Closest match among fungi (ITS sequences)		
Strain	Accession number	Genus	Most closely related strain	Ident (%)
MK-1	MK450684.1	*Penicillium* sp.	*Penicillium*	99.32%
MN-1	MN864187.1	*Penicillium* sp.	*Penicillium glaucoroseum*	100%
MG-1	MG148343.1	*Purpureocillium* sp.	*Purpureocillium*	100%

### 3.2 *In vitro* immunological activities of the fermented crude extracts of rhizosphere fungi

#### 3.2.1 Effects of fermented crude extracts on the proliferative activities of RAW 264.7 cells

**FIGURE 1:**
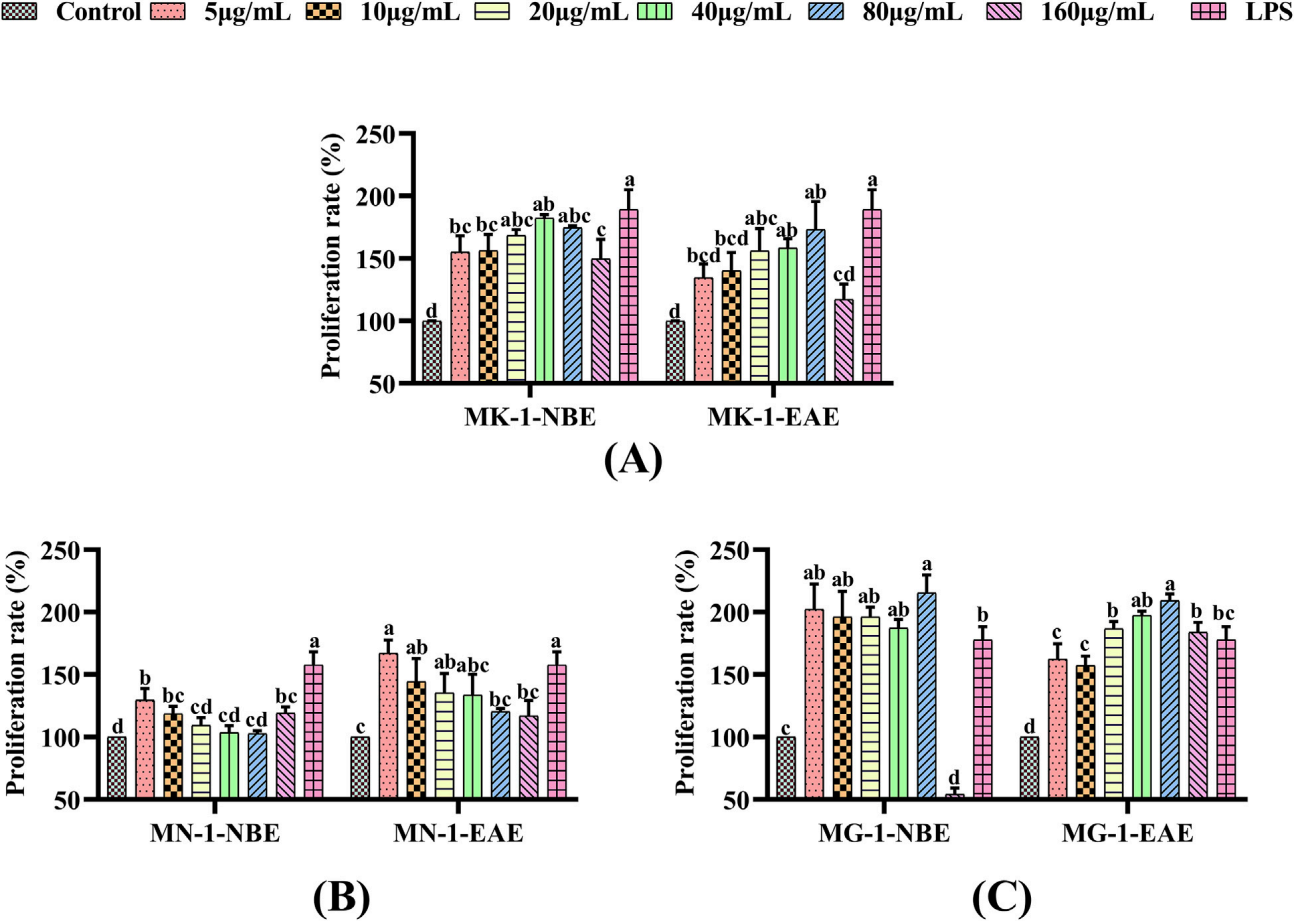
Effects of fermentation extracts of different fungi on the proliferation of RAW 264.7 cells: **(A)** MK-1; **(B)** MN-1; **(C)** MG-1. The data are expressed as mean ± SD. The different letters indicate significant differences (*p* < 0.05) among the groups.

The effects of the fermented crude extracts of the fungi on cell proliferation were evaluated through the CCK-8 assay using RAW 264.7 macrophages, whose results are shown in Figure 1. Compared to the control group, the proliferations of the macrophages were significantly enhanced by LPS and fermented crude extracts of the fungi (*p* < 0.05). As shown in Figure 1A, the proliferative effects of MK-1-NBE and MK-1-EAE tended to increase and subsequently decline with increasing concentrations, reaching maximum values at 40 μg/mL and 80 μg/mL, respectively; these showed 1.8-fold and 1.7-fold increases in proliferation compared to the control group, and there was no clear disparity with the positive control (*p* > 0.05). As shown in Figure 1B, there was a distinct difference in the proliferative activities of the macrophages following treatments with MN-1-NBE at concentrations of 5, 10, and 160 μg/mL and MN-1-EAE at concentrations of 5, 10, and 20 μg/mL over the control group (*p* < 0.05). MG-1 was observed to promote macrophage proliferation, exhibiting a significant difference in cell proliferative activity over the control group (*p* < 0.05), as shown in Figure 1C. The highest viabilities of the macrophages were observed in the treatment groups that received 80 μg/mL of MG-1-NBE and MG-1-EAE for 24 h. These levels were significantly higher than that of the LPS group, with increases of 21.09% and 17.57%, respectively (*p* < 0.05).

#### 3.2.2 Effects of fermented crude extracts on the phagocytic activities of RAW 264.7 cells

**FIGURE 2:**
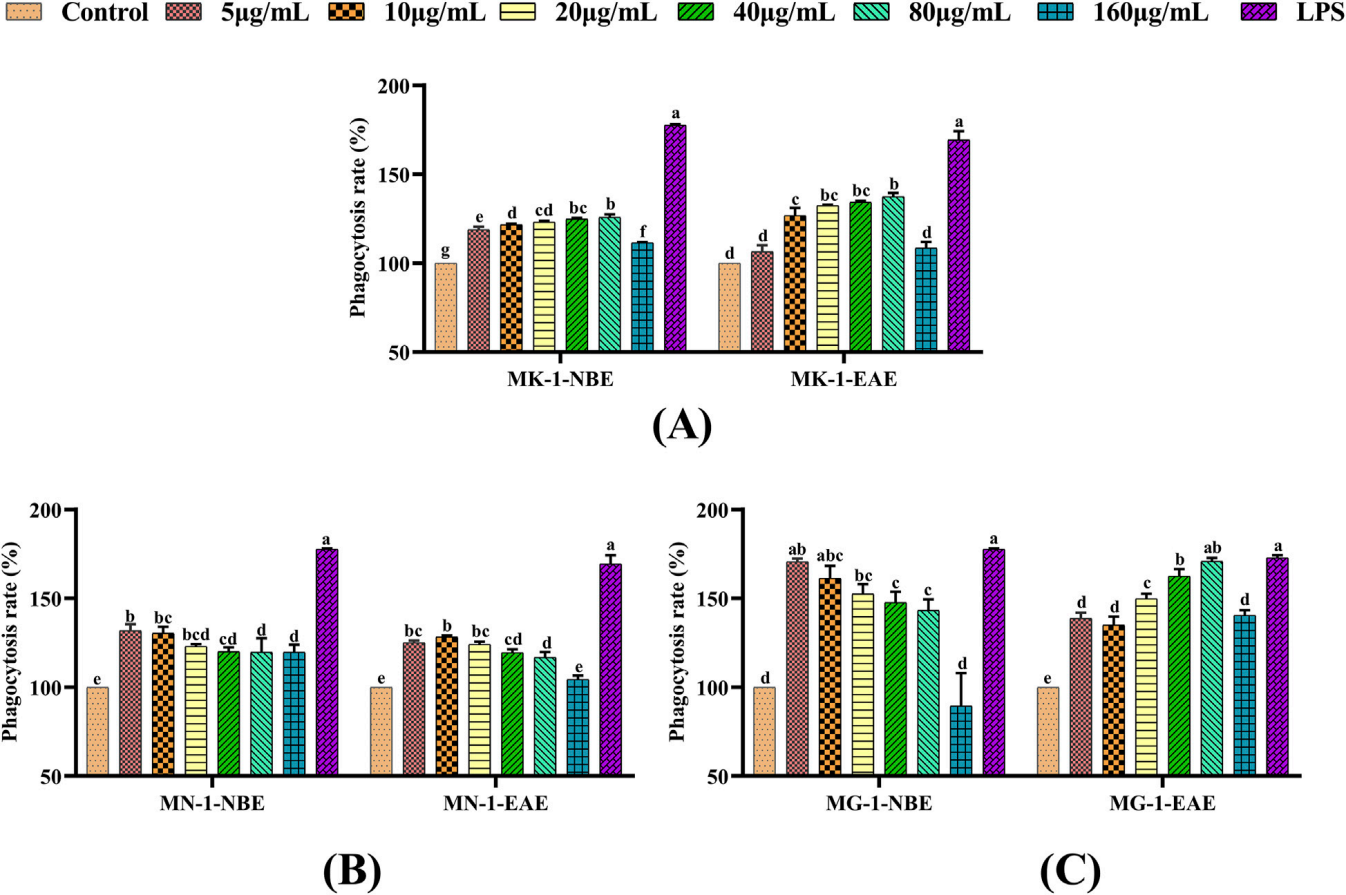
Effects of different fungal fermentation extracts on the phagocytosis ability of RAW 264.7 cells: **(A)** MK-1; **(B)** MN-1; **(C)** MG-1. The data are expressed as mean ± SD. The different letters indicate significant differences (*p* < 0.05) among the groups.

Macrophages have the capacity for uptake of the basic stain neutral red by autophagy, using which the extent of phagocytosis can be assessed. In the present experiments, the neutral red method was used to examine the effects of the fermented crude extracts on the phagocytosis of RAW 264.7 cells. These findings are illustrated in Figure 2. The NBE and EAE fractions of all three fungal fermentation products were observed to significantly enhance the phagocytosis of macrophages compared to the control group (*p* < 0.05). The phagocytosis of macrophages treated with MG-1-NBE displayed an obvious concentration-dependent trend, as shown in Figure 2C; specifically, this was a gradual decline in the capacity to promote cellular phagocytosis following treatment with MG-1-NBE at various concentrations. The maximum value of 170.6% was observed at a concentration of 5 μg/mL of MG-1-NBE, which was similar to the effect of the LPS group (*p* > 0.05). Meanwhile, after treatment with 80 μg/mL of MG-1-EAE, the phagocytosis of the macrophages reached a maximum value of 170.8%, which was not significantly different from that of the positive control (*p* > 0.05). These findings show that the fermented crude extracts of each group possessed the ability to recognize, engulf, and destroy foreign substances within the organism, in addition to enhancing the *in vitro* immunomodulatory functions of the host by promoting cellular phagocytosis. Furthermore, compared to the other groups, MG-1 demonstrated the capacity to markedly enhance cellular phagocytosis.

#### 3.2.3 Effects of fermented crude extracts on the NO contents of RAW 264.7 cells

**FIGURE 3:**
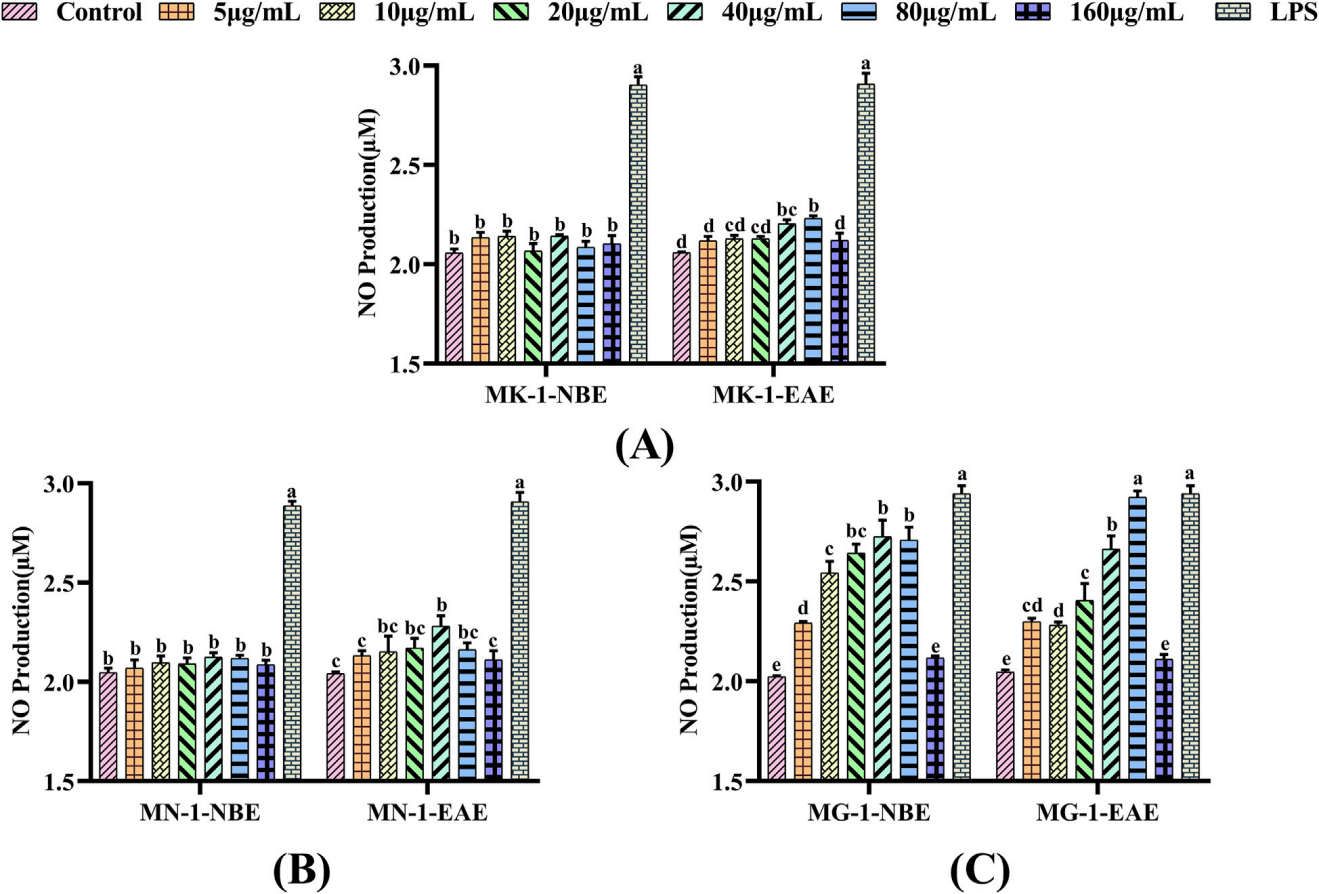
Effects of different fungal fermentation broths on the NO secretions of RAW 264.7 cells: **(A)** MK-1; **(B)** MN-1; **(C)** MG-1. The data are expressed as mean ± SD. The different letters indicate significant differences (*p* < 0.05) among the groups.

The present study examined the effects of each fungal fermentation extract on the secretion of NO in RAW 264.7 cells using the Griess method. As shown in Figure 3, a significant elevation in NO secretion was observed in the RAW 264.7 cells treated with LPS compared to the control group (*p* < 0.05). When MK-1 and MN-1 were added to the macrophages and the control group was used for comparison, only MK-1-EAE at concentrations of 40 and 80 μg/mL and MN-1-EAE at a concentration of 40 μg/mL were observed to obviously promote the release of NO from the cells (*p* < 0.05), as shown in Figures 3A, B. As seen from Figure 3C, both fractions of MG-1 exhibited pronounced effects on NO secretion compared to the control group (*p* < 0.05), which were also significant compared to the other groups of fungi, exhibiting a clear concentration dependence. When the macrophages were treated with MG-1-EAE at a concentration of 80 μg/mL, the NO content was 2.98 μM, which was essentially equivalent to that of the positive control (*p* > 0.05).

In summary, the proliferative and phagocytic activities of the RAW 264.7 cells increased after treatment with MK-1, MN-1, and MG-1 crude extracts. Compared to the control group, the NO secretions were obviously increased for treatment of the RAW 264.7 cells with both fractions of MG-1 (*p* < 0.05). It may therefore be concluded that the crude extracts of MK-1, MN-1, and MG-1 have *in vitro* immunomodulatory activities. Of these, the crude extract of MG-1 showed the best *in vitro* immunological activity, so MG-1 was identified as a highly immune-responsive fungus.

### 3.3 Effects of MG-1 crude extracts on the morphologies and cytokine secretions of RAW 264.7 cells

#### 3.3.1 Effects of MG-1 crude extracts on the morphologies of RAW 264.7 cells

**FIGURE 4:**
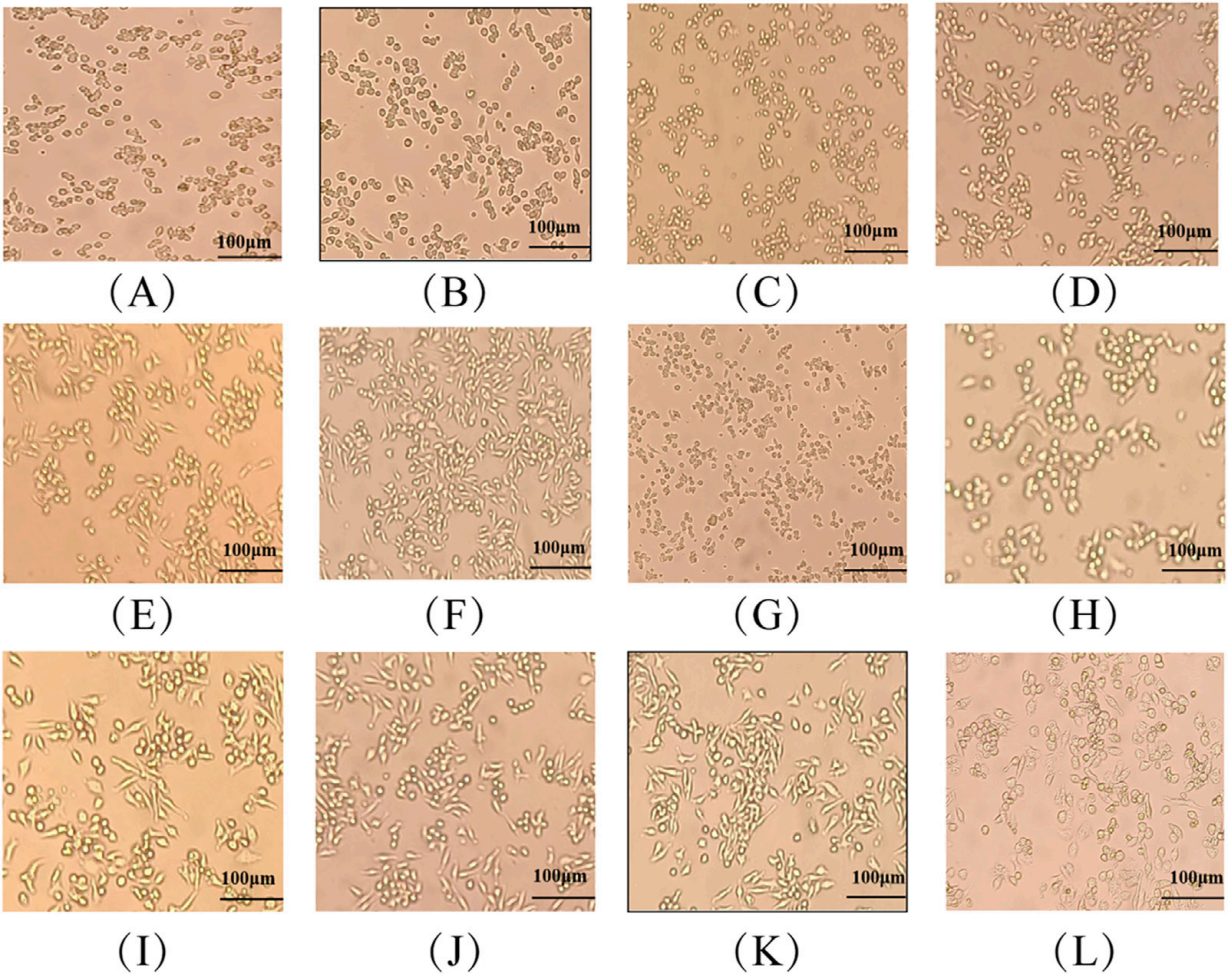
Effects of MG-1 fermentation broth on the cellular morphology of RAW 264.7 cells: **(A)** control; **(B–F)** MG-1-EAE at concentrations of 5, 10, 20, 40, and 80 μg/mL, respectively; **(G–K)** MG-1-NBE at concentrations of 5, 10, 20, 40, and 80 μg/mL, respectively; **(L)** lipopolysaccharides.

The effects of MG-1 crude extracts on the morphologies of RAW 264.7 cells are shown in Figure 4. The morphology of the cells in the control group was predominantly round and well outlined, as seen in Figure 4A. The morphological alteration to the cells in the LPS group was evident, which was characterized by increased cell volume and gradual irregular shapes as well as the appearance of more synaptic and longer spindle-shaped cells. As the concentration of the MG-1 fermentation extract increased, the cells underwent gradual transformation from round to an irregular shape. The cell volume increased, pseudopods appeared in some cells, and the tendency for cell cluster growth was evident. These results indicate that both fermentation extracts of MG-1 are capable of activating macrophages, altering cellular morphology, and enhancing the immune responses.

#### 3.3.2 Effects of MG-1 crude extracts on the production of TNF-α, IL-1β, and IL-6 in RAW 264.7 cells

**FIGURE 5:**
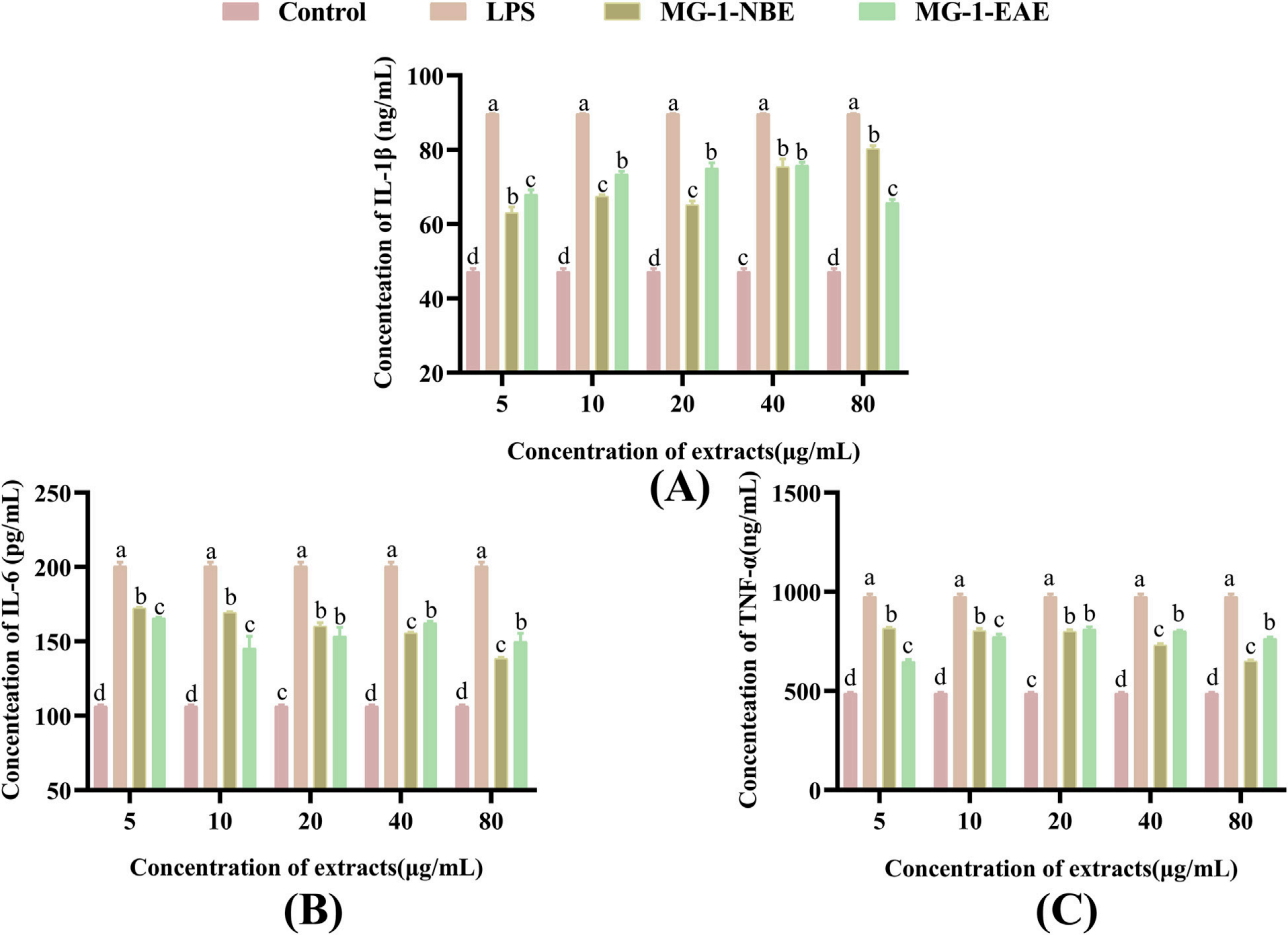
Effects of MG-1 fermentation crude extracts on the secretions of IL-1β, IL-6, and TNF-α in the RAW 264.7 cells: **(A)** IL-1β; **(B)** IL-6; **(C)** TNF-α. The data are expressed as mean ± SD. The different letters indicate significant differences (*p* < 0.05), as compared with the control group.

To evaluate the efficacies of the MG-1 crude extracts on cytokine secretions in the RAW 264.7 cells, the levels of TNF-α, IL-1β, and IL-6 were selected as the indicators for measurement. Compared to the control group, the levels of TNF-α, IL-1β, and IL-6 secreted by the macrophages were markedly elevated following treatment with LPS and the fermented crude extracts of MG-1, as illustrated in Figure 5 (*p* < 0.05). As shown in Figure 5A, there was a remarkable effect on the production of IL-1β by the macrophages following treatment with MG-1 compared to the control group (*p* < 0.05); the maximum secretion of 80.31 ng/mL was observed after treatment with 80 μg/mL of MG-1-NBE (*p* < 0.05), which was 1.7-times that of the control group. In addition, the production of cytokine IL-1β reached a maximum value of 75.42 ng/mL after treatment with 40 μg/mL of MG-1-EAE, which was 1.6-times that of the control group (*p* < 0.05). However, there was no significant difference between this treatment and the same concentration of MG-1-NBE (*p* > 0.05). The data in Figure 5B demonstrate a clear concentration-dependent trend for the secretion of IL-6 by the macrophages following treatment with varying concentrations of MG-1-NBE. The observed promotion of IL-6 secretion reached maximum values of 172.42 pg/mL and 165.68 pg/mL after treatments with MG-1-NBE and MG-1-EAE at a concentration of 5 μg/mL, respectively; these results demonstrate a 1.6-fold and 1.5-fold increase over the control group (*p* < 0.05). As illustrated in Figure 5C, treatment of the macrophages with varying concentrations of MG-1-NBE yielded a sustained concentration-dependent trend in TNF-α secretion; the secretion of the cytokine TNF-α reached a maximum value of 815.38 ng/mL, which was 1.7-fold that of the control group following treatment with MG-1-NBE at 5 μg/mL. Additionally, the secretion of TNF-α reached a maximum of 811.17 ng/mL after treatment with MG-1-EAE at a concentration of 20 μg/mL, which was 1.7-fold that of the control; this level of TNF-α secretion was not significantly different from that observed in the treatment with the same concentration of MG-1-NBE (*p* > 0.05). These results demonstrate that both of the fermented crude extracts of MG-1 significantly enhance the secretion of TNF-α, IL-1β, and IL-6 as well as regulate the immune processes in macrophage cells.

#### 3.3.3 Endotoxin contamination

To ascertain whether endotoxins had any impacts on the experimental results, the presence of endotoxins in the crude extracts of MG-1 was examined using the Gel Clot TAL Endotoxin Test kit. As shown in Table 2, the treated crude extracts of MG-1 did not exhibit a gelatinous state, thereby indicating the absence of endotoxin contamination. These results show that macrophage activation was achieved through treatment with the MG-1 crude extracts alone without the involvement of endotoxins.

**TABLE 2:** Test results of endotoxins in the crude extracts of MG-1 fermentation broth.

Sample	Positive control (LPS)		Negative control (Water)	Fermented crude extract of MG-1	
	150 μg/mL	300 μg/mL		NBE	EAE
Results	+	+	−	−	−

A positive (+) or negative (−) value indicates the corresponding presence or absence of endotoxins.

### 3.4 Identification of bioactive compounds by UHPLC-Q-TOF-MS analysis

**FIGURE 6:**
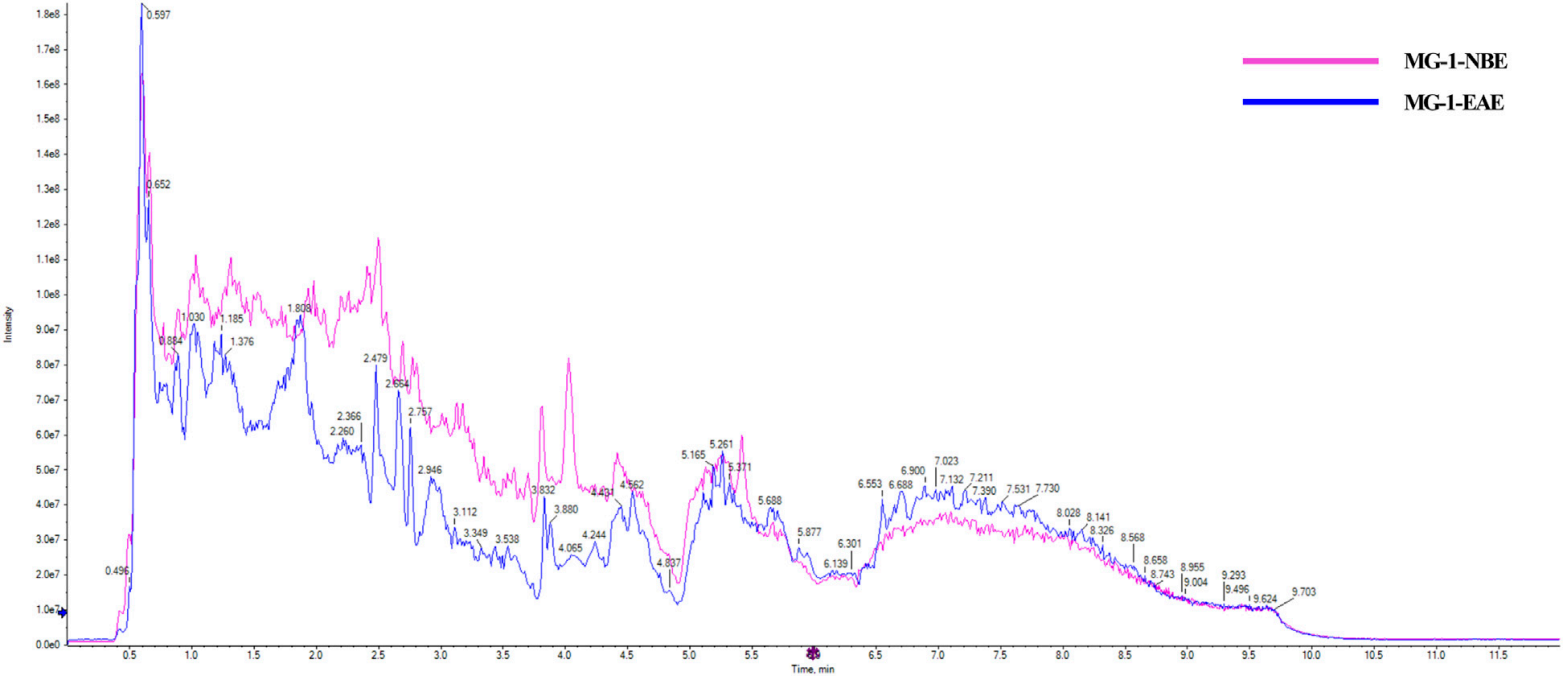
Chemical analysis of the MG-1 extracts by UHPLC-Q-TOF-MS.

Two fermented crude extracts of MG-1 demonstrating enhanced *in vitro* immunological activities were selected for further screening of bioactive metabolites. The MG-1-EAE and MG-1-EAC extracts exhibited abundant pharmacological active compounds, and UHPLC-Q-TOF-MS was selected to analyze these compounds. The resulting chromatogram is presented in Figure 6. The detected compounds were analyzed using the Agilent Masshunter Software algorithm. Only those identified compounds that ranked in the top 10% of the peak areas in at least one of the solvent extracts are shown.

The MG-1-NBE sample was found to contain several chemical compounds, including adenosine (imidazopyrimidines), menthofuran, alantolactone, artemisinin, idebenone, oleamide, carvone (prenol lipids), β-estradiol-3-benzoate (steroids and steroid derivatives), bestatin (peptidomimetics), osthole (coumarins), naltrexone (ketone compounds), quinine (cinchona alkaloids), stachydrine (carboxylic acids and derivative), L-arginine, betaine, isoprocarb, phenylethylamine (benzene and substituted derivatives), β-damascone (organooxygen compounds), 12-hydroxydodecanoic acid (hydroxy acids and derivatives), adrenosterone (steroids and steroid derivatives), and olomoucine (imidazopyrimidines). The following compounds were identified in MG-1-EAE: cyclo-prolylglycine (carboxylic acids and derivatives), daidzein, formononetin, genistein (isoflavonoids), cyclophosphamide and L-histidine (organonitrogen compounds), acetylcarnitine, prostaglandin I2 (fatty acyls), dihydrocapsaicin (phenols), deoxyguanosine (purine nucleosides), 1-deoxynojirimycin (piperidines), pyridines (pyridoxine and derivatives), 2′-o-methyladenosine, deoxyadenosine, nudifloramide (purine nucleosides), erucamide (fatty acyl group), creatinine (carboxylic acids and derivatives), and phenylacetaldehyde (benzene and substitution derivatives).

**FIGURE 7:**
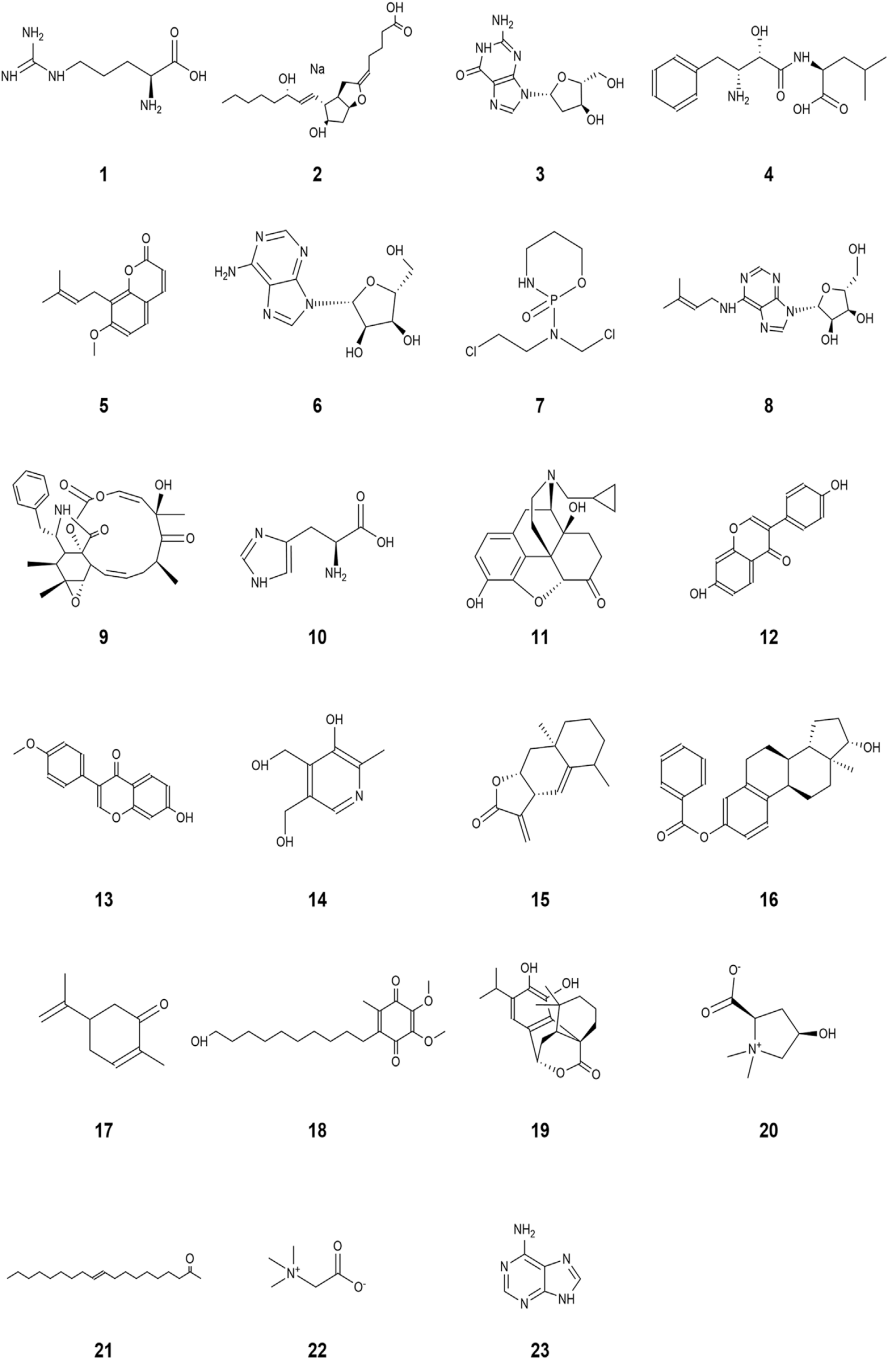
Structures with pharmacologically valuable activities identified from the bioactive components in MG-1: (1) L-arginine, (2) prostaglandin I2, (3) deoxyguanosine, (4) bestatin, (5) osthole, (6) adenosine, (7) cyclophosphamide, (8) isopentenyladenosine, (9) cytochalasin E, (10) L-histidine, (11) naltrexone, (12) daidzein, (13) formononetin, (14) pyridoxine, (15) alantolactone, (16) β-estradiol-3-benzoate, (17) carvone, (18) idebenone, (19) carnosol, (20) stachydrine, (21) oleamide, (22) betaine, and (23) adenine.

A wide range of pharmacological activities has been documented for the secondary metabolic components identified by UHPLC-Q-TOF-MS, including immunomodulatory, antitumor, antioxidant, anticarcinogenic, and antidiabetic effects. Figure 7 illustrates the chemical structures of the bioactive compounds identified from the separated fraction of MG-1, which was previously documented to possess pharmacological activity. L-arginine (compound **1**), prostaglandin I2 (compound **2**), deoxyguanosine (compound **3**), bestatin (compound **4**), and osthole (compound **5**) have been found to possess immunological activities in many prior studies. The molecular formulas, exact masses, M/Z values, and immunomodulatory activities of compounds **1**–**5** identified in the extracts are presented in Table 3. Moreover, adenosine (compound **6**), cyclophosphamide (compound **7**), isopentenyladenosine (compound **8**), cytochalasin E (compound **9**), L-histidine (compound **10**), naltrexone (compound **11**), daidzein (compound **12**), formononetin (compound **13**), pyridoxine (compound **14**), alantolactone (compound **15**), beta-estradiol-3-benzoate (compound **16**), carvone (compound **17**), idebenone (compound **18**), carnosol (compound **19**), stachydrine (compound **20**), oleamide (compound **21**), betaine (compound **22**), and adenine (compound **23**) were also identified in the extracts of MG-1. The UHPLC-Q-TOF-MS results also revealed the presence of numerous sesquiterpenoids, including artemisinin, anisatin, alantolactone, parthenolide, and cnicin. Unfortunately, the quality markers of AMR such as atractylone and lactones I, II, and III were not identified.

**TABLE 3:** Molecular formulas, exact masses, mass-to-charge ratios (M/Z), and immunomodulatory activities of the identified chemical constituents.

Compound	Molecular formula	Exact mass (Da) M/Z	Value (Da)	Immunomodulatory activity	Reference
L-arginine	C_6_H_14_N_4_O_2_	174.2	175.1	A synthetic precursor of the cytokine NO that indirectly promotes the immune efficacy of macrophages	Wu et al. (2021)
Prostaglandin I2	C_20_H_33_NaO_5_	376.47	353.2	Immunological and anti-inflammatory activities	Zeldin (2001), Dorris and Peebles (2012)
Deoxyguanosine	C_10_H_13_N_5_O_4_	267.24	152.2	Macrophages containing deoxyguanosine could induce secretions of TNF and IL-6	Davenne et al. (2019)
Bestatin	C_16_H_24_N_2_O_4_	308.37	331.2	Promotion of the secretion of interleukins 1 and 2 by macrophages	Méndez et al. (2020)
Osthole	C_15_H_16_O_3_	244.29	245.1	Antitumor, anti-inflammatory, neuroprotective, and immunomodulatory effects, as well as antibacterial activity	Zhang et al. (2015), Sun et al. (2021)

## 4 Discussion

The rhizome of *A. macrocephala* Koidz. (Compositae) from Qimen, designated as AMR, is a traditional Chinese national medicine. In “Yao Xing Lun” (A.D. 618–907), AMR is indicated for the treatment of gastric distress, alleviation of cold and fever, and stabilization of the fetus. In traditional Chinese medicine theory, these diseases are postulated to be related to immunity. Modern pharmacological studies have shown that AMR can regulate immunity. However, the resources of AMR from Qimen have declined significantly owing to slow growth and overexploitation. The plant rhizosphere was a complex ecosystem comprising interactions among numerous beneficial microorganisms (Badri et al., 2009; Butler et al., 2003). Nowadays, rhizosphere fungi with pharmacologically active effects and their secondary metabolites have been found to be present in large quantities in medicinal plants (Pan et al., 2021; Shankar and Sharma, 2022). In a previous study, we found that there were significant differences in the composition, diversity, and functionality of the rhizosphere microbial community between the AMR from Qimen and captive AMR. The AMRs from Qimen and their rhizosphere fungi have formed a highly exclusive symbiotic mechanism, demonstrating specificity across different varieties of the same plant. As a consequence of long-term synergistic evolution, the metabolites produced by these rhizosphere fungi were found to be similar to those of the host plant. This allowed us to hypothesize that the fermented crude extracts of the rhizosphere fungi of AMR could also have immunomodulatory effects.

In this study, MK-1, MN-1, and MG-1 were isolated from the rhizosphere soil of the AMR from Qimen, which were identified as belonging to the *Penicillium* sp. and *Purpureocillium* sp. The *Penicillium* sp. is a common microscopic filamentous fungus found in soil, cereals, and air (Demjanová et al., 2020). Furthermore, its metabolites exhibit a range of activities, including antibiotic, antiviral, and mycotoxic properties (Demjanová et al., 2020). *Purpureocillium* sp. is a common soil fungus that plays a role in controlling various pathogens and insects, thereby maintaining the balance of the soil ecosystem (Lin et al., 2016). Meanwhile, a previous study revealed that exopolysaccharides derived from the fungus *Purpureocillium* sp. have immunomodulatory capabilities (He et al., 2019).

Immunity is the ability of a host to defend against various pathogens in the form of bacteria, viruses, and parasites. Macrophages are known to be important immune cells that play key roles in immune responses (Mantovani and Sica, 2010; Mortezaee and Majidpoor, 2022; Zhang and Wang, 2014) and are activated to eliminate pathogens and tumor cells from the body (Mantovani and Sica, 2010). Macrophage are effective defense cells in an organism, and their phagocytosis is an important non-specific type of immune response (Lin et al., 2018; Li et al., 2017). Macrophages undergo a process of renewal and replenishment within an organism, which are often achieved by continual proliferation (Liao et al., 2015). In certain instances, certain pathogenic microorganisms and foreign bodies in an organism can be cleared non-specifically by activated macrophages through phagocytosis and chemotaxis (Hirayama et al., 2017; Rubio et al., 2018). NO as the primary mediator of macrophage resistance to bacterial and tumor cell invasion maintains the immune homeostasis of the body, so it can be regarded as a criterion for determining macrophage activation (Zhang et al., 2020; Tümer et al., 2013). NO levels have been demonstrated to exert immunomodulatory functions within the body and also combat tumors and pathogenic microorganisms (Vannini et al., 2015). Consequently, RAW 264.7 cells were used to study the *in vitro* immune activities of the fermented crude extracts of the rhizosphere fungi of AMR. The *in vitro* immunomodulatory activities of the fermented crude extracts of different rhizosphere fungi of AMR were initially evaluated through their cell proliferation capacity, cell phagocytosis activity, and NO secretion capacity. LPS is a popular endotoxin that has the capacity to stimulate macrophages and exert immunomodulatory effects (Xiao et al., 2019); thus, it was selected as the positive control in this study. As illustrated in Figures 1–3, the crude extracts of MK-1, MN-1, and MG-1 increase the proliferative and phagocytic activities of the RAW 264.7 cells. Furthermore, the crude extracts of MK-1-EAE, MN-1-EAE, and MG-1 enhance NO secretion in the RAW 264.7 cells. It is well-known that appropriate proportions of immune factors could improve the ability of an organism to resist external stimuli and repair damage. Conversely, overproduction of immune-active substances can cause inflammatory responses in the cells, leading to cellular damage and apoptosis (Giacoppo et al., 2017). Here, the *in vitro* immunological activity of MG-1-EAE was found to be more pronounced than that of the NBE fraction. It has been revealed that the extraction efficiencies of compounds mainly depend on the pH, temperature, sample–solvent ratio, and solvent polarity (Al Obaydi et al., 2020). Ethyl acetate is a neutral solvent and is favored for the extraction of phenolic compounds, such as the isoflavone daidzein (Sujono et al., 2021), phenolics (Abd-Alla et al., 2017), alkaloids (Baban et al., 2023), flavonoids, and ellagic acids (de Araújo et al., 2024), which have been reported to exhibit strong immunological activities. In conclusion, MK-1, MN-1, and MG-1 crude extracts all show *in vitro* immunomodulatory activities. In this study, MG-1 was screened further for its high immunological activity.

In their resting state, macrophages typically exhibit round or oval shapes. Following stimulation of the macrophages with an activator, a series of typical morphological changes are observed. The cells exhibit a notable increase in size, a flattening of their character, and the development of a large number of cellular protrusions (Sun et al., 2023). These cellular protrusions may facilitate the engulfment and digestion of pathogens as well as cellular waste by the macrophages (Sun et al., 2023). As the concentration of MG-1 fermentation extract is increased, the cells undergo a gradual transformation from round to an irregular shape. The cell volume increased, pseudopods emerged in some cells, and the tendency for cell cluster growth became evident, which also indicates that the process could indeed activate macrophages (Figure 4). IL-1β participates in pain, inflammatory responses, and autoimmune processes within the body; it is highly involved in the cellular defenses and tissue repair processes of an organism (Mauer et al., 2014). IL-6 is produced by the immune cells as a cytokine to regulate the functional roles of leukocytes and differentiation of B cells (Mauer et al., 2014). TNF-α levels have been linked to the ability of the macrophages to take up substances and resistance of the lymphocytes to external pathogenic microorganisms (Hennessy et al., 2017). As shown in Figure 5, both of the fermented crude extracts of MG-1 and LPS significantly promoted secretions of the cytokines IL-6, IL-1β, and TNF-α to regulate the immune processes in RAW 264.7 cells. The modulation of IL-6, IL-1β, and TNF-α production may have contributed to the immunomodulatory effects of MG-1, which is consistent with the findings of previous studies examining the effects of IL-6, IL-1β, and TNF-α on the evolution of innate immunity during inflammation through elimination of abnormal cells (Samodova and Dobrodeeva, 2012; Zhu et al., 2016; Hu et al., 2019). To ascertain whether endotoxins exerted any influences on the experimental results of this study, the presence of endotoxins in the crude extracts of MG-1 was examined; the results showed that macrophage activation was achieved through treatment with the MG-1 fermentation crude extract alone without the involvement of any endotoxins in the cellular activation process. Therefore, we concluded that MG-1 has pronounced immunomodulatory effects *in vitro*.

The ethyl acetate and n-butanol extracts of MG-1 displayed considerable pharmacological activities and were thus subjected to further UHPLC-Q-TOF-MS analysis. The biological activities of the fungal fermented crude extracts may be mainly associated with previously identified compounds having documented pharmacological effects, although these could be obtained through different routes. The two extracts of MG-1 were found to contain various bioactive substances, including L-arginine, prostaglandin I2, deoxyguanosine, bestatin, and osthole. These substances were demonstrated to exert immune functions through disparate mechanisms. As a precursor for the synthesis of NO and the only amino acid substrate for NO production by all NOS isoforms, L-arginine plays a pivotal role in regulating NO production (Wu et al., 2021). It has been shown that T regulatory cells, which help maintain the body’s immune homeostasis, may be obviously facilitated by prostaglandin I2 in terms of production and action benefits (Dorris and Peebles, 2012; Zeldin, 2001; Norlander and Peebles, 2021). Treatment of bone-marrow-derived macrophages with deoxyguanosine has been shown to exert immunomodulatory effects through the increased secretion of TNF and IL-6 (Davenne et al., 2019). As a superior immunomodulator, bestatin obviously affects the functions of T-lymphocytes in mice by improving the levels of interleukins 1 and 2 (Méndez et al., 2020). Osthole has been shown to play non-negligible and important roles in antitumor, anti-inflammatory, neuroprotective, immunomodulatory, and antimicrobial applications given its rich range of biological activities (Sun et al., 2021; Zhang et al., 2015). Adenosine has been proven to exert antitumor immune effects by relying on four different adenosine receptors (Kazemi et al., 2017). Lower concentrations of naltrexone have been observed to elicit antitumor effects, particularly through interference with cell signaling and modulation of the immune system (Liu and Dalgleish, 2022).

In addition to the immune-boosting active ingredients mentioned above, secondary metabolites with wide ranges of pharmacological activities were identified, including antitumor, antioxidant, anticarcinogenic, and antidiabetic effects. Many studies have confirmed that immunomodulatory functions have close relationships with other pharmacological activities (Slovak et al., 2017). For example, antitumor immunity is a prerequisite for successful cancer intervention (Sun et al., 2016). The extracts of MG-1 were found to contain adenosine, cyclophosphamide, isopentenyladenosine, cytochalasin E, L-histidine, and naltrexone, which have been demonstrated to elicit substantial antitumor effects. Cyclophosphamide is a widely used chemotherapeutic agent for the treatment of various malignant tumors (Ahlmann and Hempel, 2016). As a phytochelatin essential for the binding of filamentous fibrillar-tRNA to messenger RNA, isopentenyladenosine has been shown to have significant antitumor activity (Kazemi et al., 2017). Glycoalkaloids such as solanidine derived from Solanum plants are reported to have antitumor activity against various tumor cell lines (Korpan et al., 2004). As a fungal metabolite, cytochalasin E was observed to significantly inhibit proliferation and phagocytosis in A549 cells, thereby offering a potential opportunity for the development of therapeutic agents against lung cancer (Takanezawa et al., 2018; Takanezawa et al., 2017). L-histidine has the potential to mitigate the toxic effects of anticancer drugs and significantly improved the therapeutic efficacies of several anticancer substances in a mouse tumor model (Elshafie et al., 2023). Naltrexone had been shown to modulate the immune system at various concentrations, thereby exerting indirect antitumor effects (Liu and Dalgleish, 2022). Daidzein, formononetin, pyridoxine, alantolactone, beta-estradiol-3-benzoate, carvone, idebenone, carnosol, and stachydrine also exhibit potent antioxidant properties. Most of the flavonoids are also found to exhibit antioxidant properties; flavonoids such as soya sapogenins, formononetin, and others found in the MG-1 extracts also have this ability (Laddha and Kulkarni, 2023; Ma and Wang, 2022). The pharmacological effects of pyridoxine are numerous and diverse; its profound antioxidant and antimicrobial abilities play significant roles in protein reconstitution, neurotransmitter biosynthesis, and other activities (Sharma et al., 2023). Studies have shown that alantolactone exhibits antitumor, antibacterial, and anti-inflammatory properties; it is a chemical component isolated from the rhizome of the late tuberose (Cai et al., 2021). As a free radical scavenger, beta-estradiol-3-benzoate demonstrates the ability to inhibit lipid peroxidation (Aguirre-Vidal et al., 2017). Furthermore, carvone exhibits excellent antioxidant effects and is a frequent naturally occurring substance in agricultural and medicinal applications (Chen et al., 2021; Decarvalho and Dafonseca, 2006; Souza et al., 2013; De Sousa et al., 2007). Idebenone, carnosol, and stachydrine have been identified as key agents in antioxidant therapy (Jaber and Polster, 2014; Chun et al., 2014; Liao et al., 2023). Oleamide, betaine, and adenine have been shown to exert anti-inflammatory activities; oleamide enhances LPS-induced phagocytosis of the microglia *in vivo* and *in vitro* and also exhibits significant anti-inflammatory activity as a dual-active material (Jo et al., 2015). Betaine has been shown to exert anti-inflammatory activities in various diseases, and positive effects have been reported in the treatment of many fatty liver diseases and diabetes mellitus (Jung et al., 2013; Kathirvel et al., 2010; Kim et al., 2017). Adenine has been shown to exert anti-inflammatory effects in inflammatory cellular models, particularly in the treatment of inflammatory bone disease (Chen et al., 2020). In reality, the presence of multiple biomolecules in the extracts of MG-1 that are effective against different pathways of immunomodulatory activities could enhance the innate immunomodulatory capabilities through synergistic mechanisms. Given the demonstrated immunomodulatory potentials of the extracts in this study, there is need for further investigation. To the best of our knowledge, the present study is a pioneering work on the *in vitro* immunomodulatory activities of rhizosphere fungal extracts derived from a medicinal plant that provides a novel opportunity for studying safe and stable immunomodulators.

## 5 Conclusion

The findings of the present study indicate that MK-1, MN-1, and MG-1 isolated from the rhizosphere fungi of AMR exhibit significant *in vitro* immunomodulatory activities. The crude extracts of MG-1 were comparatively found to have the highest levels of *in vitro* immunomodulatory activities, as evidenced by their ability to enhance the proliferative capacity, promote phagocytosis, and secrete multiple cytokines of RAW 264.7 cells. L-arginine, prostaglandin I2, deoxyguanosine, bestatin, osthole, and other bioactive molecules were identified in the extracts of MG-1 by UHPLC-Q-TOF-MS and have been reported to exhibit immunological activities in many studies. It was postulated that these bioactive components were the reason for the excellent immunomodulatory abilities exhibited by the two MG-1 extracts in this study. We propose that MG-1 might represent a novel biological resource with clinical applications capable of activating macrophages. To the best of our knowledge, this is a pilot comparative study of the *in vitro* immunomodulatory activities of different solvent extracts derived from the rhizosphere fungi of medicinal plants, demonstrating their beneficial immunomodulatory effects and roles in the recognition of bioactive substances. The present work provides new resources for the development of immune-active substances and offers a valuable approach for further exploitation of rhizosphere fungal resources. The present study also offers preliminary insights into the immunomodulatory effects of MG-1 *in vitro*. However, further studies are necessary to develop immunologically active substances for broader usage.

## Data Availability

The raw data supporting the conclusions of this article will be made available by the authors without undue reservation.
